# Automated and Manual Quantification of Tumour Cellularity in Digital Slides for Tumour Burden Assessment

**DOI:** 10.1038/s41598-019-50568-4

**Published:** 2019-10-01

**Authors:** Shazia Akbar, Mohammad Peikari, Sherine Salama, Azadeh Yazdan Panah, Sharon Nofech-Mozes, Anne L. Martel

**Affiliations:** 1 Physical Sciences, Sunnybrook Research Institute, Toronto, Canada; 2 Medical Biophysics, University of Toronto, Toronto, Canada; 3 Vector Institute, Toronto, Canada; 4 Sunnybrook Health Sciences Centre, Toronto, Canada

**Keywords:** Breast cancer, Translational research, Breast cancer

## Abstract

The residual cancer burden index is an important quantitative measure used for assessing treatment response following neoadjuvant therapy for breast cancer. It has shown to be predictive of overall survival and is composed of two key metrics: qualitative assessment of lymph nodes and the percentage of invasive or *in situ* tumour cellularity (TC) in the tumour bed (TB). Currently, TC is assessed through eye-balling of routine histopathology slides estimating the proportion of tumour cells within the TB. With the advances in production of digitized slides and increasing availability of slide scanners in pathology laboratories, there is potential to measure TC using automated algorithms with greater precision and accuracy. We describe two methods for automated TC scoring: 1) a traditional approach to image analysis development whereby we mimic the pathologists’ workflow, and 2) a recent development in artificial intelligence in which features are learned automatically in deep neural networks using image data alone. We show strong agreements between automated and manual analysis of digital slides. Agreements between our trained deep neural networks and experts in this study (0.82) approach the inter-rater agreements between pathologists (0.89). We also reveal properties that are captured when we apply deep neural network to whole slide images, and discuss the potential of using such visualisations to improve upon TC assessment in the future.

## Introduction

Neoadjuvant systemic therapy (NAT) for breast cancer (BC) is used to treat locally advanced and operable BC to allow breast-conserving surgery^1^. NAT provides an opportunity to monitor clinical, radiological and ultimately pathologic response. Pathologic complete response (pCR) to NAT has been shown to provide accurate surrogate endpoint for patient survival^2^ and in some studies has been prognostic for rate of local recurrence^3^. As such, accurate assessment of pathologic response to NAT provides important prognostic information.

As residual disease can be subtle and/or scattered, the assessment of pCR to neoadjuvant therapy relies upon a stable and standardized protocol applicable across multiple institutions. Symmans *et al*.^4^ proposed a method for quantifying residual disease by calculating the residual cancer burden index (RCB). RCB is recognized as long-term prognostic utility^4^ and has shown to be more predictive of overall survival compared to other measurements^5^. The RCB index accounts for two key metrics: qualitative assessment of residual disease in the breast (via tumour cellularity (TC) in the tumour bed (TB) and proportion of *in situ* component) and assessment of lymph nodes. Here, the TB area comprises of morphologic changes in the stroma suggestive of tumor regression with or without residual invasive or *in situ* carcinoma. While RCB calculator produces a continuous score, scores are further categorized in four RCB classes from pCR (RCB-0) to extensive residual disease (RCB-III) that are easily reproducible^6^. Accurate quantification of TC is a laborious, time consuming task that most practicing pathologists are not trained to perform. Yet, TC is crucial for computing the RCB index.

Currently TC is estimated by manually “eyeballing” the TB area at multiple microscopic fields through several slides that represent the largest linear dimension, and comparing the involved area with graphic standard sketches^7^. Such illustrations although helpful are semi-quantitative, subjective measurements. A single case-level score is then obtained by taking an average of TC scores from different fields and rounded to the nearest 10th percent. However, these scores can also be reformulated on a continuous scale. As manual analysis is limited to predefined discrete values, we are yet to discover the potential benefits of continuous TC scores for prognosis. In theory such measurements can be more precise, however, acquiring them manually is infeasible given there is a greater chance of error and reproducibility is not possible.

**Figure 1:**
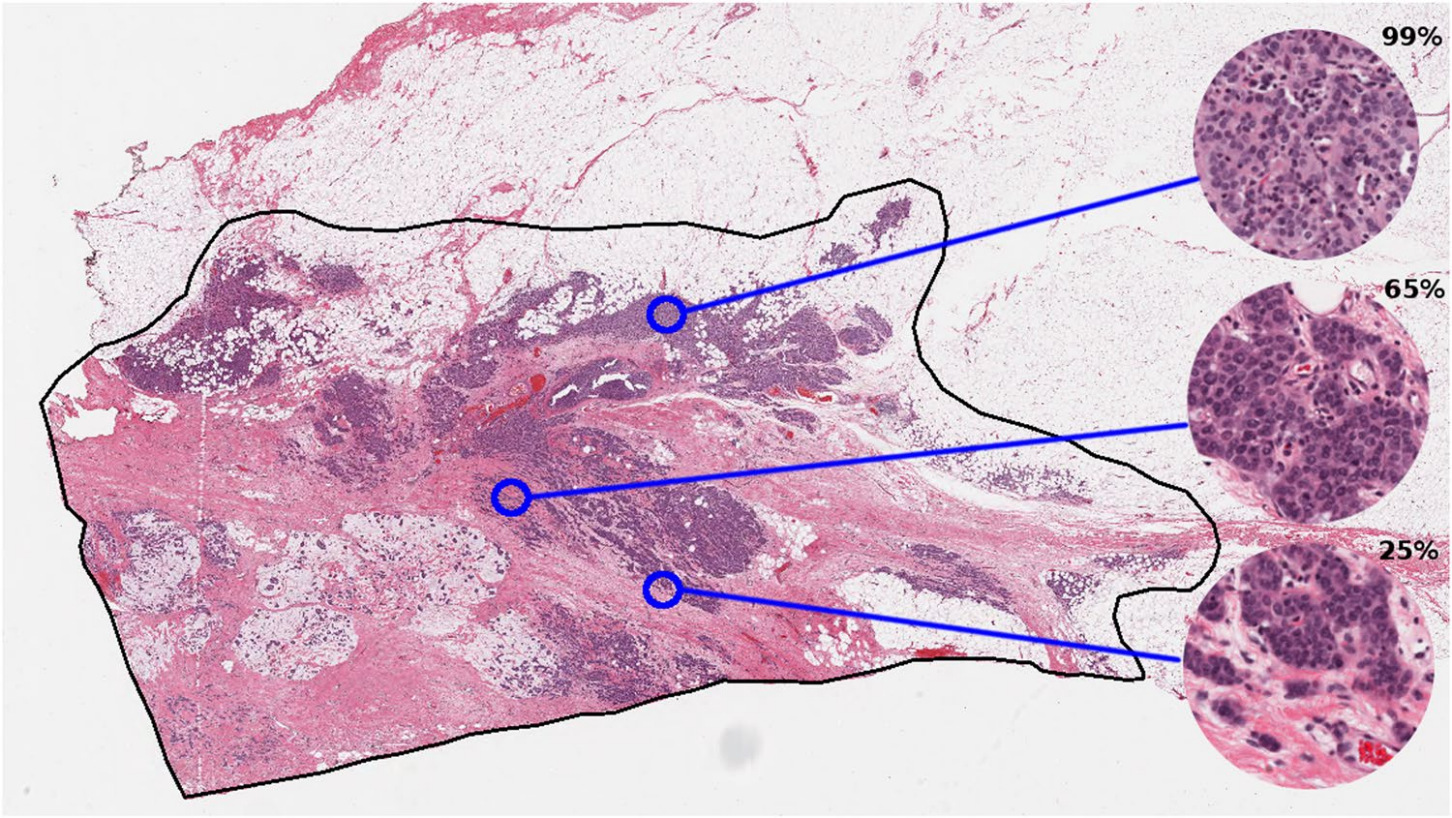
TB outlined in black in a digital slide scanned at 20X magnification (displayed at lower resolution). Regions of interest are shown in a higher magnification on the right alongside TC scores provided by an expert pathologist.

**Figure 2:**
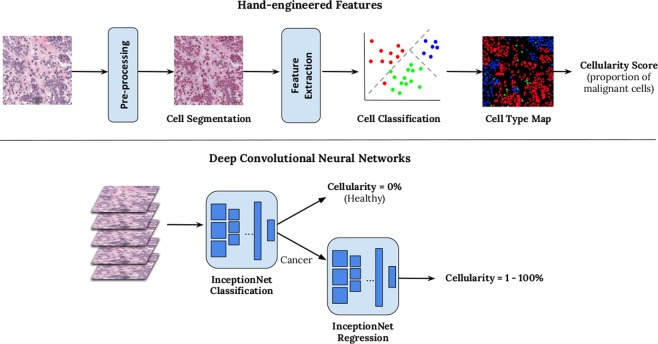
Overview of two methods for generating automated TC score. Hand-engineered feature approach is shown above and a cascade approach using two deep convolutional neural networks below.

With latest technological advancement in digital pathology, including tissue scanners capable of scanning whole slides at high resolutions, there is potential to leverage image analysis techniques to gain more accurate metrics than is achievable by the human eye, and reduce pathology workload by eliminating time-consuming tasks. TB region can be captured digitally on scanned slides, using annotation tools (Fig. 1). Towards an ultimate goal to automate RCB assessment, we explored methods to evaluate TC as the first step. In this paper, we report the use of automation to compute TC scores using two different image analysis approaches (Fig. 2):

1. An image analysis pipeline which encompasses hand-engineered features designed to mimic the pathologist’s eye.
2. A deep learning approach which learns features directly from raw image data of digital slides using deep convolutional neural networks (DCNNs).

We performed detailed comparisons between the above approaches to validate the feasibility of automation in the pathology workflow and measure the progression of image analysis techniques over the last few years to perform RCB assessment, which currently relies heavily on expert opinion. In this study we define TC as the percentage of total area within predefined areas of interest or patches, occupied by malignant tumour cells. The tumour area defined in Symmans *et al*.^4^ excludes tumour infiltrating lymphocytes (TILS). Pathologists, however, will also include areas of cytoplasm surrounding malignant nuclei in their cellularity assessment and this is a somewhat subjective process. Whilst automation can also be used to locate the TB, in this paper we specifically report computation of TC with predefined boundaries, with the intention of implementing this as part of a larger pipeline in future work.

In this paper, we report the benefits and limitations of latest advancements in artificial intelligence, compared to a more traditional hand-engineered approach for designing algorithms. To evaluate the clinical relevance of automation on whole slide images, we also applied our trained models to high resolution digital slides scanned at 20X, achievable in a matter of minutes. We show that a localised analysis of TC on a patch-by-patch basis can be used to give a more descriptive representation of the heterogeneity of the TB area and distribution of TC scores.

## Results

### Agreements between manual and automated scores

A quantitative comparison between two pathologists: a pathology fellow (Pathologist A) and an experienced breast pathologist (Pathologist B) and our two proposed image analysis pipelines described in Section 4 are given in Table 1 in the form of intra-class correlation coefficient (ICC) values. ICC values are reported between patches i.e. regions of interest measuring 258 *μm* × 258 *μm*, extracted from digital slides. The reported intra-rater agreement between the study pathologists was 0.89 and this value indicates the level of variability between our readers in the independent test set. Both automated methods fell short of reported intra-rater agreements however automatically-generated scores were more consistent as shown by reported confidence intervals (shown in square brackets). Such outcomes suggests that automated TC scores are consistently stable with scores retrieved from both annotators, demonstrating the advantage of reproducibility with such systems. Out of the two automated methods, DCNNs were superior, with an average agreement of 0.82, close to agreements between our experts and substantially higher than hand-engineered features. Given the upper and lower bounds of reported scores, DCNNs were on par with inter-rater agreements, and furthermore are reproducible.

**Table 1:** Two-way intra-class correlation (ICC) coefficients between two pathologists, and two automated methods for predicting TC scores.

	ICC Coefficient (95% CI)			
	Pathologist A	Hand-engineered^12^	DCNN^8^	Combined
Pathologist A	—	0.74 [0.70, 0.77]	**0.83** [0.79, 0.86]	0.76 [0.74, 0.79]
Pathologist B	0.89 [0.70, 0.95]	0.75 [0.71, 0.79]	**0.81** [0.78, 0.84]	0.79 [0.76, 0.81]

Upper and lower bounds are given in square brackets.

To further evaluate DCNN, we also combined both automated systems by identifying cancerous patches from the first InceptionNet model in DCNN (Fig. 2) and then using hand-engineered features to determine an automated TC score. Results are shown on the right in Table 1. Whilst ICC values are better than hand-engineered features alone, DCNN is still superior suggesting both InceptionNets are needed for optimal performance.

### Comparison between hand-engineered features and deep learning

A breakdown of prediction accuracies between both automated systems revealed that the DCNN trained to solely distinguish between health and cancerous tissue performed exceptionally well, giving accuracy rates of 93% when compared against both experts. Our hand-engineered approach fell short at 81% due to mis-identified malignant cell nuclei during the cell classification stage.

**Figure 3:**
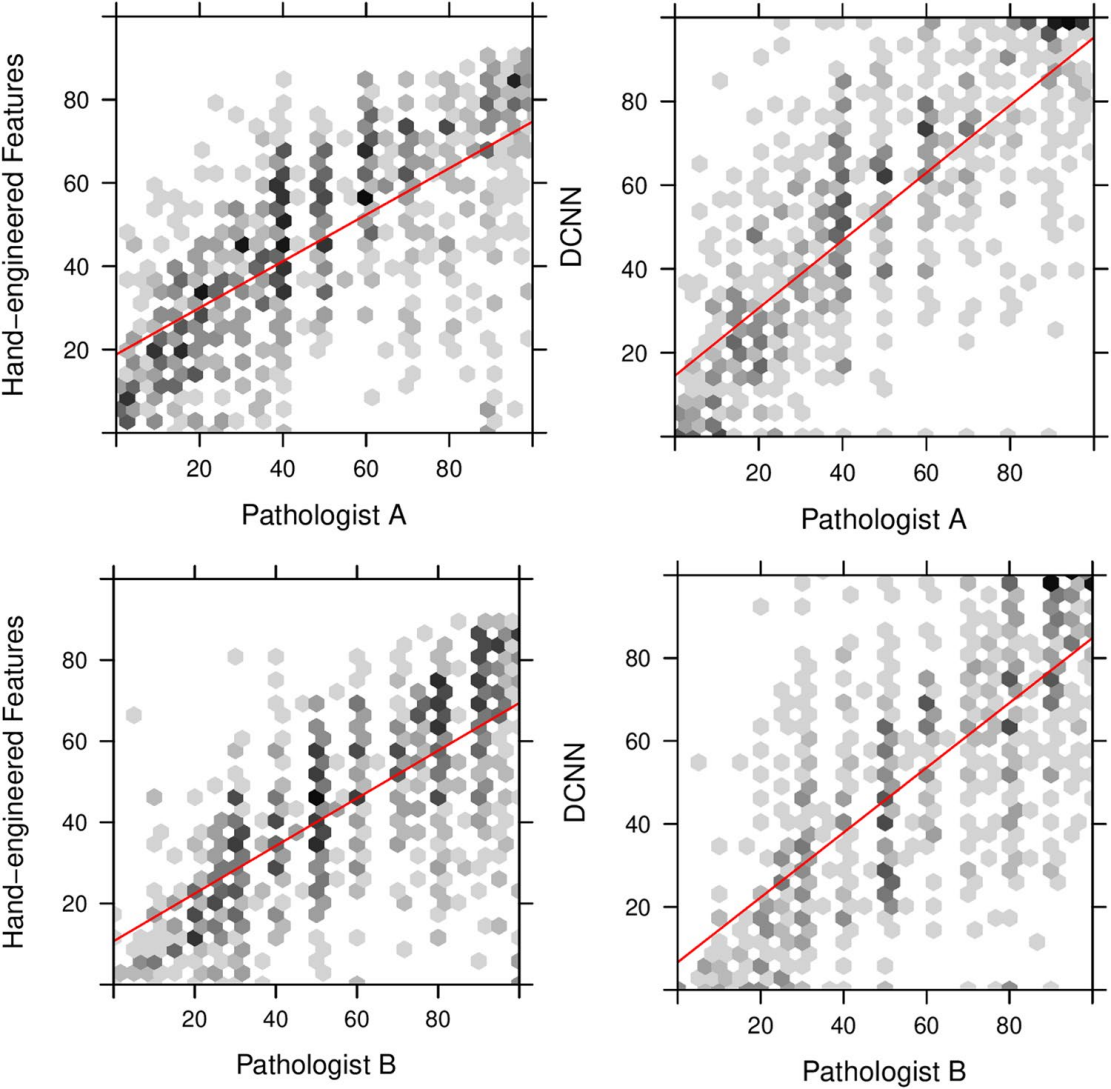
TC scores between 0% and 100% predicted by a hand-engineered approach (top) and deep neural networks (bottom) against scores provided by both expert pathologists (Pathologist A, Pathologist B).

**Figure 4:**
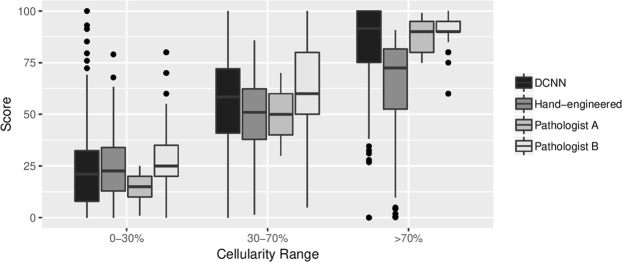
Boxplot of distribution of scores within low (0-30%), medium (30-70%) and high (>70%) ranges of TC.

When comparing TC scores for those patches which contained few cancerous structures i.e. TC scores just above 0%, we found the hand-engineered approach produced cellularity scores with strong concordance with expert pathologists (Fig. 3 (left)). Whilst DCNN generated cellularity scores with good agreement with our study experts, as shown by the line of best fit, generally scores were not as precise as the hand-engineered approach. However the lack of outliers, particularly in the 0–30% range, meant that DCNN performed the best overall. The hand-engineered approach particularly suffered in the >70% range as shown in Fig. 4, suggesting further work is needed to represent regions containing high proportions of tumour cells.

## Discussion

In this paper, we evaluated three methods for generating TC scores on digital slides of breast tissue for the purposes of tumour burden assessment. The standard method for computing TC scores is by visual interpretation of the TB which is a time consuming process and is limited to a rough estimate of the proportion of cancerous structures in an irregular region of interest performed by an expert. Furthermore, manual analysis is subject to inter- and intra-rater variability therefore reproducibility of TC scores is a limitation in current practice.

To increase throughput, we designed two alternative methods for generating TC scores which leverage advancements in technology to automatically analyse large whole slide images. One approach was to mimic the way in which a pathologist would compute a score, by first identifying cells in a given region of interest and then measuring the proportion of malignant to benign cells and stroma. This approach has been well adopted in the digital pathology community which has led to a large literature on cell classification and segmentation^8–10^ and feature extraction methods^11^.

In the last five years there has been a shift in medical image analysis to automatically extract features from image data alone using deep architectures^12^. The advantages of this approach is that there is no hand-engineering of features involved, and instead appropriate image properties are captured in a model containing several layers. There has been previous work using deep neural networks in digital pathology^13,14^, and comparisons have shown we can achieve superior performance compared to traditional feature extraction methods^15–17^. In this study, we also found that by using deep neural networks, we could achieve strong agreements with scores produced by two study pathologists; achieving ICC agreements of 0.82, approaching the intra-rater agreement of 0.89 and with tighter upper and lower bounds, suggesting more stable measurements than can be achieved manually. Our hand-engineered approach fell short at 0.75 ICC agreement. Given these outcomes, there is potential to use automation to alleviate the burden of manually estimating TC scores which would allow assessments such as the RCB index more manageable on a routine basis. Furthermore, our method can be used across multiple tumour types for TC assessment with some fine-tuning.

**Figure 5:**
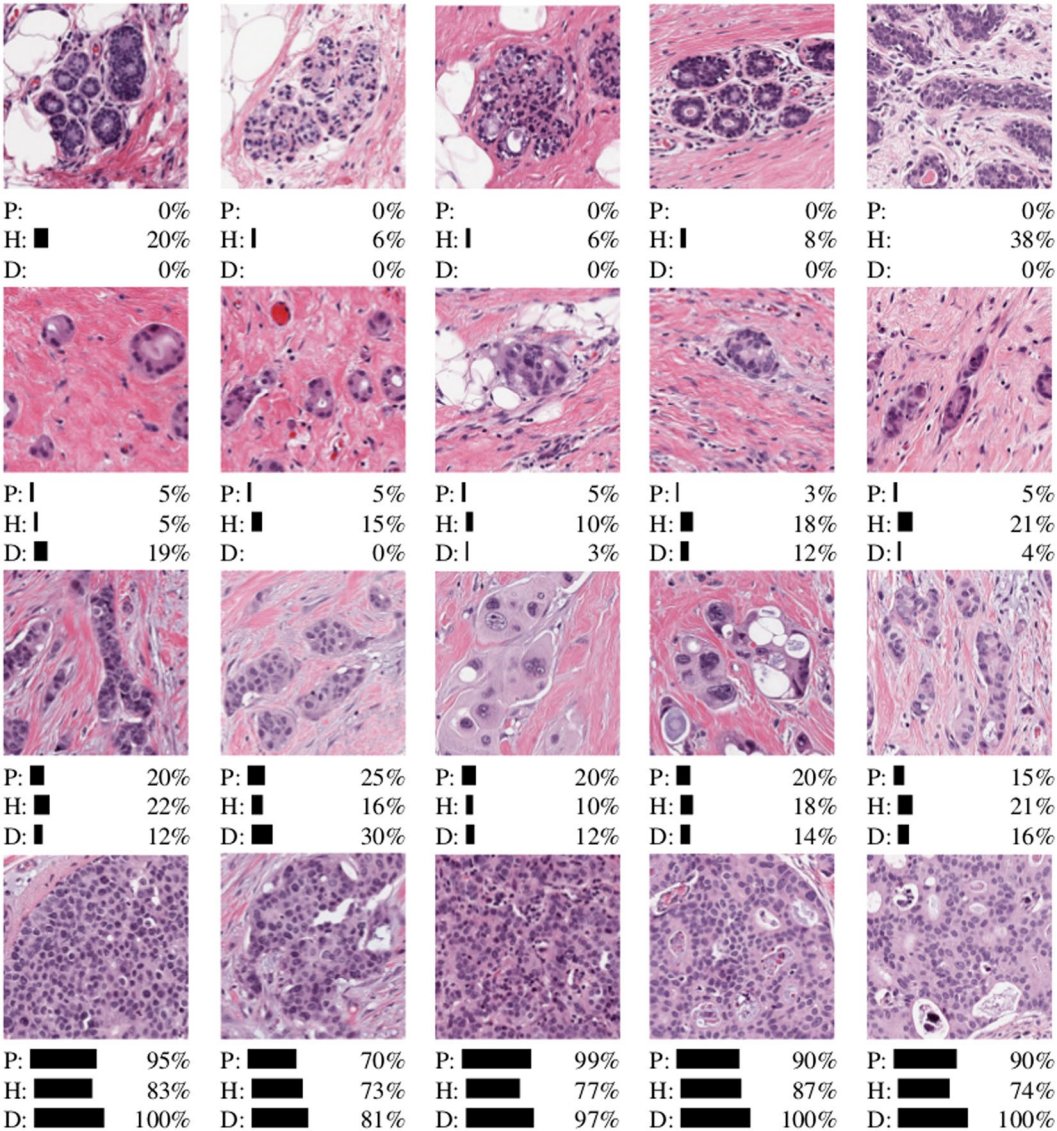
Subset of results from TC test dataset for healthy tissue, and low/medium/high TC categories (top to bottom). Scores are given for both automated systems (H = hand-engineered features, D = deep convolutional neural networks) and Pathologist A (P).

Upon closer inspection of our results, we also found under certain conditions the use of latest automated techniques produced TC scores more similar to our experts. A subset of the scores produced by both automated systems are shown in Fig. 5. The deep neural networks (D) performed better when identifying healthy tissue (top row) and patches containing almost all cancerous tissue (bottom row). The cascade approach we adopted of training a separate cancer detector, proved to be ideal for removing healthy tissue first, giving accuracy rates of 93% when identifying patches containing only healthy structures. The advantage of the deep learning approach comes in distinguishing between >70% TC, and this is demonstrated in Fig. 3, whereby automated scores are tighter around those scores assigned manually.

For the purposes of determining RCB, accurate quantification of TC in the lower range leading to RCB-0 and RCB-1 classes, may enhance clinical prognostication by adopting automation. Symmans *et al*.^4^ reported RCB-1 was a good predictor of survival outcome with 89% of triple negative breast cancer patients with RCB-1 relapse-free after 5 years; RCB-2 and RCB-3 were not prognostic. Given that the deep neural networks excelled at low TC range, the use of automation for measuring TC as a RCB component could potentially be improved by adding further training examples containing low TC scores. As it is particularly difficult to quantify TC manually, automation offers an easier method for achieving precise scores which can further contribute to use of the RCB index as opposed to RCB categorical readings.

It should be noted that whilst both automated methods reported here output scores on a continuous scale, the scores provided to the systems during training were not. Manual assessment was performed by providing an estimate of the proportion of carcinoma in each patch, often to the nearest 5% in our experiments; some variations between automation and pathologists’ scores can be explained by the scoring protocol.

Whilst here we specifically evaluated TC, the RCB index also encompasses a measure of the TB area^7^. Assessment of TB size relies upon consideration of preNAT imaging, gross examination and expert interpretation of the TB. In the current study, TC was assessed in patches derived from predefined TB regions, determined by Pathologist A. As such, this work is only an initial step in automating the entire RCB calculation pipeline. Further work is needed to identify “TB” and to distinguish between invasive and *in situ* carcinoma in a fully automated pipeline. This may require assessment of multiple digital slides per patient. Furthermore, we were only able to obtain scores from two raters; this is an expensive and time consuming process and it was not possible to recruit more pathologists to carry out this task. Another limitation is that all slides were prepared and scanned at the same centre; a secondary test set from another institution would allow generalizability to be assessed.

**Figure 6:**
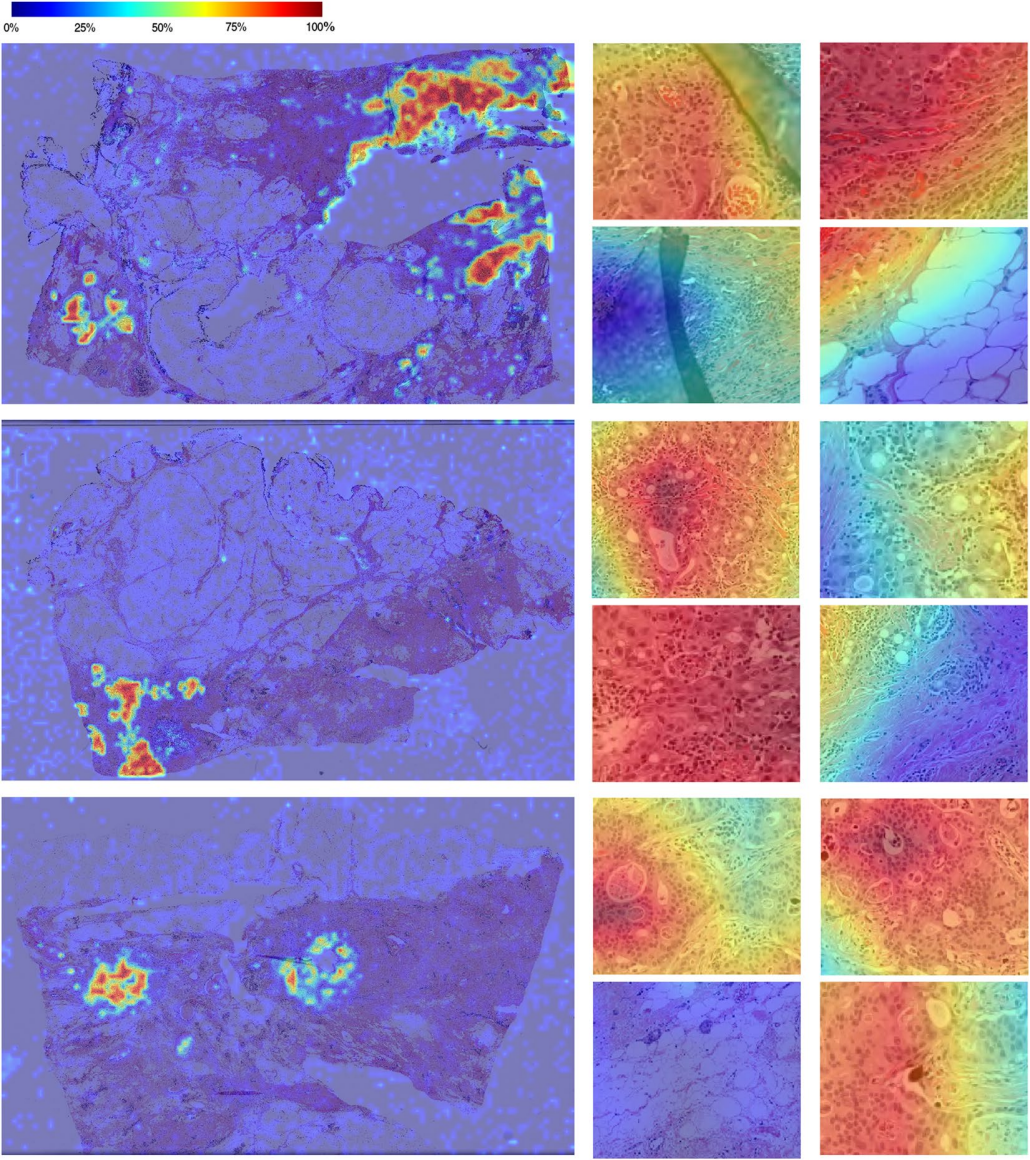
TC scores produced by a trained deep neural network overlaid on whole slide images. Scores are provided on a patch-by-patch level, where blue denotes healthy (0% TC) and red denotes 100% TC. Some close-up results of cellularity scores are provided to the right of each whole slide image.

One of the main advantages of using automation is the ability to perform detailed analysis across entire whole slide images to give further contextual information. An example of our trained deep neural networks applied to whole slide images is shown in Fig. 6, as heatmaps overlaid on original digital slides. We have appended a higher resolution image as Supplementary Fig. S2. Blue overlays denotes low TC and red denotes patches with high TC scores. In its simplest form, this tool can be used to navigate the reader to the most interesting parts of the tissue thus eliminating around 90% of the slide. This is a desirable property in digital pathology as the substantial portion of a pathologists’ time is performed sifting through benign tissue^18^ and any method for increasing throughput has significant advantage in the pathology workflow. It is important to note that the deep neural network designed to distinguish between healthy and cancerous patches suffered when applied to whole slide images compared to our patch-based test set. During training, the model was only exposed to a small subset of healthy structures and as such fatty tissue, folding tissue, red blood cells etc. were therefore unrecognizable during testing. We anticipate that with further training with more healthy patches, such errors can be avoided. In the long term, a preprocessing phase to first identify the TB region is recommended.

In Fig. 6, we can also see a distinct distribution of TC scores across the TB, suggesting that a global score of the entire TB may not reflect the characteristics of the TB accurately. There are clearly “pockets” of high cellularity regions and most of the TB consists of healthy or low cellularity regions. The RCB index recommends recording the average TC, however our results suggests an alternative metric which takes into account spatial distribution of TC in the TB may offer new features possibly advantageous for assessing tumour burden. Further work is also needed to investigate using continuous scores for clinical assessment, specifically the relationship between heterogeneity of the TB and prognosis.

To summarise, we performed a comparison between manual and automated assessment of TC and showed that we can gain reproducible scores with automation, and superior performance with deep neural networks. We showed that leveraging such tools on whole slide images can give us richer representations of tumour heterogeneity across the TB, and can potentially be used as an alternative metric to approximated TC scores currently used in practice.

## Methods

### Data

To validate different methods for computing TC, representative sections from 62 patients with residual invasive BC on resection specimens following NAT were acquired. After de-identification, representative routine Hematoxylin and Eosin glass slides were scanned at 20X magnification (0.5 lm/pixel) in the Department of Anatomic Pathology at Sunnybrook Health Sciences Centre (SBHSC), Toronto, Canada. After initial assessment and quality control, nine patients in our study were excluded. Table 2 shows a summary of patients’ clinical characteristics in this study.

**Table 2:** Clinical pathology characteristics of patients in reported study.

Criteria		Total
Age at Diagnosis	30–39	12
	40–49	17
	50–59	11
	60–72	13
Histology	Invasive ductal carcinoma (IDC)	50
	Invasive lobular carcinoma (ILC)	4
	Invasive mammary carcinoma (IMC)	1
Grade	1	8
	2	29
	3	15
ER	Positive	37
	Negative	16
PR	Positive	30
	Negative	22
HER2	Positive	11
	Negative	42

Note that multiple WSIs were prepared per patient, and therefore one patient may share multiple characteristics.

This study was approved by the Research Ethics Board (REB) of Sunnybrook Health Sciences Centre (project ID: 312 2014) and all methods were carried out in accordance with relevant guidelines and regulations. Informed consent was waived by the REB of SBHSC. The distribution of our training and test sets were such that patient data used for training was excluded from testing. Patches, each with dimensions 512 × 512 pixels, were hand-selected from 96 whole slide images, 25 of which were reserved for testing purposes. Patches were selected to represent a wide representation of range of TC scores. In total we extracted 3,700 patches (training: 2579, test: 1121) which were then labelled manually.

### Manual interpretation

For this study, we recruited two pathology experts: a breast pathology fellow (Pathologist A) and an experienced pathologist with focused practice in breast pathology (Pathology B). Each pathologist independently annotated identified patches on a digital pathology viewing platform, Sedeen Viewer (Pathcore)^19^. Initially, Pathologist A was asked to select representative patches spanning a full range of TC scores (i.e. from low to high TC) as well as patches containing healthy tissue. For each patch, a TC score, ranging from 0% to 100% for assessment of RCB^7^, was provided. Patches which did not contain any tumour cells were assigned a TC score of 0%. The training set was only annotated by Pathology A. Both Pathologist A (the annotator for the training set) and Pathologist B annotated the test set, and we compared the variability between both experts in reported results. In addition to continuous scores, both pathologists also classified each patch to low, medium, high TC and no tumour cells. Annotations were performed independently therefore each expert was unaware of scores assigned by the other.

The distribution of TC scores provided by each pathologist is given in Table 3. Note that the distribution varies considerably at higher cellularity scores (i.e. >70%) within our test sets, with 18% of Pathologist A’s scores within this range, and 31% in Pathologist B’s scores. Any automated system must be able to adjust for these differences between our experts.

**Table 3:** Number of patches in training and test set which fall into each TC score range.

	TC Score Range			
	0%	1–30%	31–70%	>70%
Train (Pathologist A)	701	840	665	373
Test (Pathologist A)	242	225	301	353
Test (Pathologist B)	237	312	375	197

### Hand-engineered features

To mimic scores provided by pathologists in an automated manner, we first designed a cell nuclei segmentation algorithm to identify boundaries of individual cells of the following types: lymphocytes, epithelial cells, malignant cells. Cells boundaries were identified by removing stain variations through a series of color stretch and color space conversions. A support vector machine (SVM) classifier was trained from several appearance, textural and spatial features extracted from identified cell nuclei, producing a cell map during testing (Fig. 2 (top)).

To compute a cellularity score, we segmented all malignant nuclei in each patch, dilated the resulting binary mask to account for the presence of cytoplasm, and then computed the fraction of area covered by malignant tissue. Finally, a calibration step was performed to bring computed TC scores closer to scores manually assigned by the pathologists in our study. A full description of this algorithm is given by Peikari *et al*.^20^.

### Deep convolutional neural network

Deep convolutional neural networks (DCNNs) are a family of architectures in artificial intelligence which are derived from a conceptual model of the human brain^12^. Typically, a DCNN consists of multiple layers, each of which contain several artificial neurons. By learning connections between hundreds or even millions of these neurons through simple linear activation functions, we can capture representations of complex data inputs. In a DCNN, groups of neurons are stacked in a series of specialised layers which can model further abstract representations of the data without manual intervention and this is where the power of DCNNs lie. Whilst there are many approaches to building DCNNs, here we opted to finetune a prebuilt network called InceptionNet^21^ which has been well-adopted in digital pathology. To compute TC scores, we trained two separate InceptionNets: one that distinguished between healthy and cancerous tissue, and the other to output regression scores on a continuous scale between 0% and 100%. Details of the implementation of the InceptionNet models is provided in supplementary material (see Supplementary Data S1).

### Software and tools

Algorithms to produce hand-engineered features were developed in Matlab and the built-in “fitcsvm” library was used to produce SVM predictions. The DCNN system was implemented in Python and Keras with a TensorFlow backend, a well-adopted library developed by Google for building deep neural networks. In each platform, we developed functions to load manual annotations generated by the Sedeen Viewer (see Section 4.2).

R was used to perform statistical analysis in this study.

## Supplementary information

SP1 DCNN Description

SP2 Whole Slide Images High Res
