# Supplementary material for: Automated and Manual Quantification of Tumour Cellularity in Digital Slides for Tumour Burden Assessment
Source: Sci Rep. 2019 Oct 1;9:14099. doi: 10.1038/s41598-019-50568-4 (PMC6773948; doi:10.1038/s41598-019-50568-4)
Supplement: Supplementary file 2 — SP2 Whole Slide Images High Res [file 41598_2019_50568_MOESM2_ESM.pdf]

Automated and Manual Quantification of Tumour Cellularity in Digital Slides for Tumour Burden Assessment

Shazia Akbar[1,2,3], Mohammad Peikari[2], Sherine Salama[4], Azadeh Y. Panah[4], Sharon Nofech-Mozes[4], and Anne L. Martel[1,2,3]

[1]Physical Sciences, Sunnybrook Research Institute, Toronto, Canada

[2]Medical Biophysics, University of Toronto, Toronto, Canada

[3]Vector Institute, Toronto, Canada

[4]Sunnybrook Health Sciences Centre, Toronto, Canada

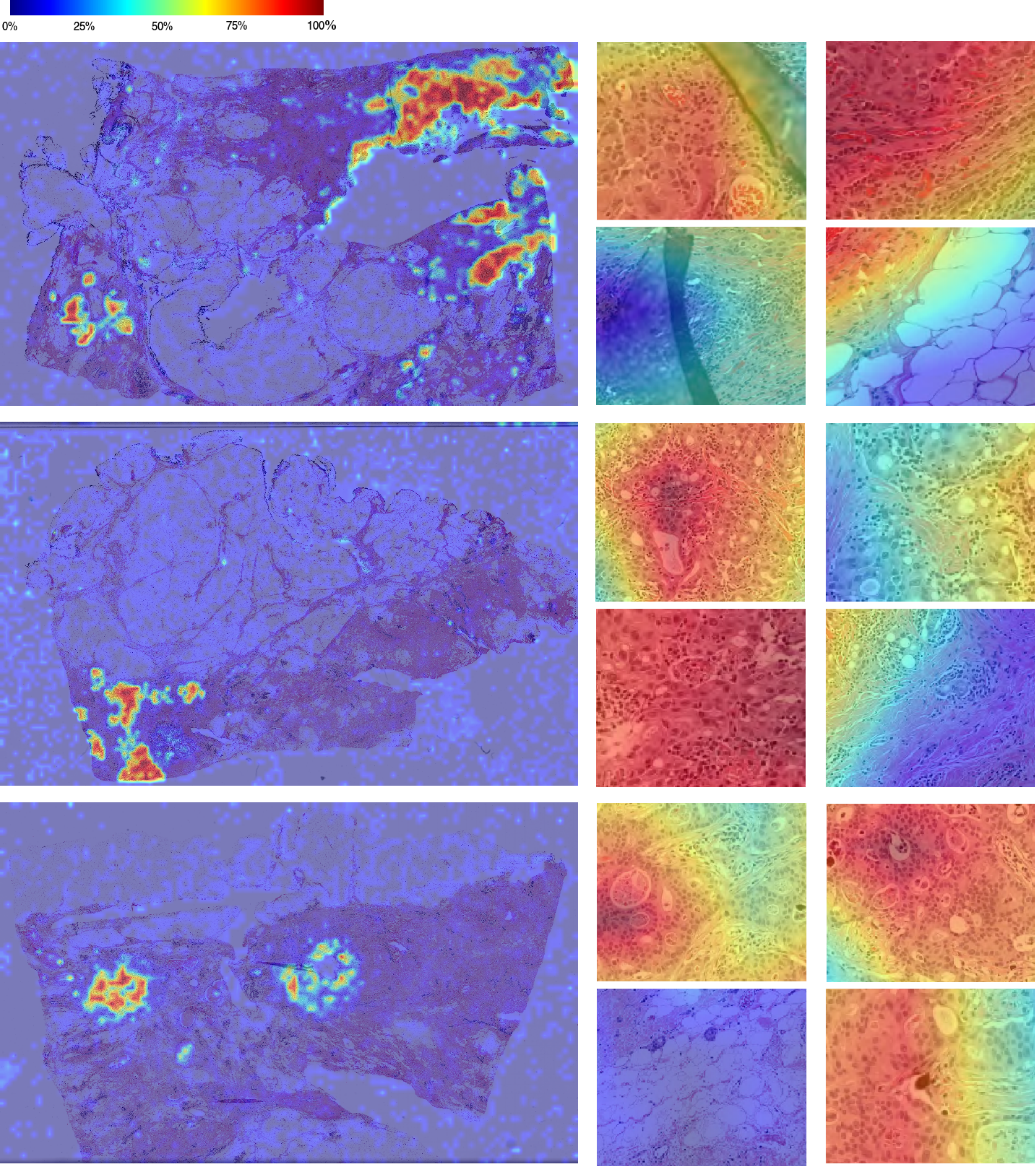
0%
25%
50%
75%
100%
